# Recurrent peritonitis relapse in a patient with atrial septal defect undergoing peritoneal dialysis: a case report

**DOI:** 10.1186/s12882-022-03037-6

**Published:** 2022-12-16

**Authors:** Jianying Wang, Shengqin Wu, Jingshu Sun

**Affiliations:** Weifang People’s Hospital, No. 151 Guangwen Street, Kuiwen District, Weifang, 261000 China

**Keywords:** Peritoneal dialysis (PD), Relapsing peritonitis, Infective endocarditis (IE), Atrial septal defect (ASD), Case report

## Abstract

**Background:**

Peritonitis is the most common complication in patients undergoing peritoneal dialysis (PD). Most patients recover with appropriate antibiotic treatment; however, when peritonitis repeatedly relapses, the cause of recurrence must be explored. The relationship between atrial septal defect (ASD), infective endocarditis (IE), and peritonitis is rarely reported. Here, we present a case of recurrent peritonitis due to *Staphylococcus aureus* in a patient with ASD and IE undergoing PD.

**Case presentation:**

A 46-year-old woman with chronic renal failure secondary to chronic glomerulonephritis experienced three episodes of peritonitis within 80 days of starting PD. The patient had a history of untreated ASD without symptoms. After undergoing PD for approximately 35 days, the patient was admitted to our hospital on April 5, 2016, due to abdominal pain and fever for 1 week (maximum temperature of 38.5 °C) accompanied by chills and shivering. The PD effluent from the time of her admission was positive for *S. aureus*. Thereafter, peritonitis recurred each month. When the third episode of peritonitis occurred, transthoracic echocardiography was performed, and a vegetation measuring 9.5 × 6.4 mm attached to the surface of the right ventricle around the ventricular septal membrane was identified. Finally, the patient was diagnosed with IE. Then, ASD repair surgery was successfully performed after the infection was controlled. The patient was followed up for 5 years, with no further episodes of recurrence.

**Conclusions:**

When a patient with ASD undergoing PD develops peritonitis, especially relapsing peritonitis, the possibility of IE is significantly increased. ASD repair surgery may be an important contributing factor to prevent peritonitis recurrence.

## Background

Peritonitis is one of the most common complications in patients undergoing peritoneal dialysis (PD), and it is associated with high morbidity and mortality rates [1, 2]. In Hong Kong, > 16% of deaths in patients treated with PD occur secondary to peritonitis [3]. Similarly, 18% of cases of infection-related mortality in patients undergoing PD result from peritonitis in the United States [4].

*Staphylococcus aureus* (*S. aureus)* is one of the most common pathogens associated with peritonitis [5, 6]. Most patients recover from peritonitis with appropriate antibiotic treatment. However, when peritonitis relapses repeatedly, the cause of recurrence needs to be explored.

Here, we report a rare case of relapsing peritonitis in a patient with atrial septal defect (ASD) and infective endocarditis (IE). Peritonitis recurred three times before the diagnosis of IE was confirmed. The patient was successfully treated with intraperitoneal and intravenous antibiotic therapy and ASD repair surgery.

## Case presentation

On February 28, 2016, a 46-year-old Chinese woman with end-stage renal disease secondary to chronic glomerulonephritis visited our hospital due to severe nausea and vomiting, and she requested to undergo PD. The patient’s past medical history included chronic glomerulonephritis for 8 years; chronic renal dysfunction and renal hypertension for 4 years, for which she was taking amlodipine to control blood pressure; untreated congenital heart disease without symptoms; and appendectomy performed 30 years prior.

The patient’s physical examination on admission revealed the following characteristics: height, 160 cm; weight, 52 kg; body temperature, 36.5 °C; heart rate, 74 beats/min; respiratory rate, 18 breaths/min; blood pressure, 144/77 mmHg. She had mild eyelid edema, pale eyelid conjunctiva, and mild lower-extremity edema. All other examination results were unremarkable.

The full blood count revealed a white blood cell count of 5.41 × 10^9^/L with 70.2% neutrophils, a red blood cell count of 2.86 × 10^12^/L, a hemoglobin concentration of 82 g/L, and a platelet count of 182 × 10^9^/L. The blood test results revealed an albumin concentration of 49 g/L, a blood urea nitrogen concentration of 41.4 mmol/L, a creatinine concentration of 1119 μmol/L, an estimated glomerular filtration rate of 4.54 mL/min, and a C-reactive protein concentration of 4 mg/L. Transthoracic echocardiography revealed three secundum ASDs, two of which were located near the superior vena cava (measuring 5.4 mm and 5.2 mm, respectively) and the other near the inferior vena cava (measuring 7.6 mm). All ASDs had left-to-right shunts, as well as minor mitral regurgitation and slight right atrial enlargement. No other concomitant disorders were detected, such as ventricular septal defect, aortic or mitral valve disease, or patent ductus arteriosus.

Due to the high creatinine concentration, the patient required urgent dialysis. On the third day of hospitalization, the straight, double-cuffed Tenckhoff 41-cm silicon PD catheter was implanted by open surgery. The patient was administered intravenous cefazolin (0.5 g) before the procedure for antibiotic prophylaxis. After PD catheter implantation, the urgent-start PD regimen was initiated. Immediately, the dialysis prescription was administered, with a fill volume of 1000 mL and a dwell time of 4 h (four times per day for 5 days). Then, the PD regimen was changed to continuous ambulatory PD (CAPD) with three 2-L exchanges of 1.5% dextrose PD solution (Dianeal PD4, Baxter) per day. The Baxter double-bag system was used, and glucose solution served as the penetrant. The dressing was changed on the third day after surgery, and suture removal was performed 10 days after catheter implantation. The patient was educated on the PD technique on the third day after catheter insertion by a trained PD nurse. After 12 days of treatment, symptoms of uremia were eliminated, and the patient was discharged. The patient continued CAPD at home with a fill volume dialysate exchange of 2 L three times per day.

The patient was re-admitted to the hospital on April 5, 2016, due to abdominal pain and fever for 1 week (maximum temperature of 38.5 °C) accompanied by chills and shivering. The patient’s physical examination revealed the following characteristics: blood pressure, 149/95 mmHg; body temperature, 37.6℃; normal cardiopulmonary physical examination; and abdominal muscle tension, tenderness, and rebound pain. The PD catheter exit site was normal, and there was no swelling or pain around the tunnel. The PD effluent was turbid, with a white blood cell count of 1900/μL and a neutrophil percentage of 87%. The patient was diagnosed with PD-related peritonitis.

According to 2016 International Society for Peritoneal Dialysis (ISPD) guidelines [7], the patient underwent empirical antibiotic treatment with cephalothin (1 g/day) and amikacin (200 mg/day) administered intraperitoneally. On the fourth day of hospitalization, the patient’s symptoms improved and her body temperature decreased to within the normal range. The PD effluent became clear, and the white blood cell count of the effluent decreased to 40/μL with mononuclear leukocyte predominance. Simultaneously, we discovered that the PD effluent culture from the time of her admission was positive for *S. aureus*. Although blood cultures were not obtained, antibacterial treatment was adjusted according to the PD effluent culture results. We discontinued cephalothin and started intermittent vancomycin (1.5 g every 5 days for 3 weeks), and amikacin was discontinued after 7 days to protect residual renal function. In the meantime, tunnel ultrasonography was performed to rule out tunnel infection. The exit site was dry without exudation. There were no manifestations of inflammation, and because antibiotic therapy had been started, nasal swab and catheter exit site culture were not performed.

On May 13, 2016, the patient was readmitted with the same symptoms and signs as the previous episode. The PD effluent was turbid, with a white blood cell count of 1920/μL and a neutrophil percentage of 80%. Computed tomography of the heart, chest, abdomen, and pelvis showed no sign of infection. The cultures of the catheter exit site and nares were negative. The PD effluent was positive for *S. aureus*, which is susceptible to vancomycin and amikacin. Although blood cultures were also not obtained this time, we did not identify any other potential sources of infection. Therefore, we speculate that the initial anti-infective treatment duration was not long enough. When the patient was admitted to the hospital, vancomycin and amikacin were selected as the antibiotics for empirical treatment. The PD effluent turned clear after 2 days, and the white blood cell count of the effluent was normal after 4 days. Therefore, we did not change the antibiotic regimen. The patient was treated with amikacin (200 mg/day) for 1 week, but intermittent vancomycin (1.5 g every 5 days) was used for 4 consecutive weeks intraperitoneally.

On June 21, 2016, peritonitis relapsed again. This time, we performed PD effluent culture and blood culture simultaneously. The PD effluent culture and the two sets of blood cultures revealed the presence of the same organism, *S. aureus*. Transthoracic echocardiography was performed again, but this time, a vegetation measuring 9.5 × 6.4 mm attached to the surface of the right ventricle around the ventricular septal membrane was identified. Even though the white blood cell count of the PD effluent was normal on the sixth day of hospitalization, intermittent vancomycin (1.5 g) was administered intraperitoneally every 5 days for 4 weeks, while piperacillin and tazobactam (2.25 g every 8 h) were administered intravenously for 3 weeks until the vegetation was no longer detected on transthoracic echocardiography. To avoid further peritonitis relapse, ASD repair surgery was successfully performed by open heart surgery at Beijing Anzhen Hospital affiliated to Capital Medical University. No vegetations were found on the surface of the right ventricle during surgery. The patient has thus far been followed up for 5 years, with no further episodes of recurrence.

## Discussion

In this study, we report a case of recurrent peritonitis relapse caused by *S. aureus* associated with IE. *S. aureus* is both a commensal bacterium and a human pathogenic microorganism. In fact, approximately 30% of the human population has been colonized by *S. aureus* [8], which is the leading cause of bacteremia and IE, as well as osteoarticular, skin, soft tissue, pleuropulmonary, and device-related infections.

CAPD is commonly used to treat patients with end-stage renal disease. Peritonitis is a serious complication of PD [9–11], especially peritonitis caused by *S. aureus*, and it is associated with poor outcomes and high PD failure rates. *S. aureus*-related peritonitis is generally the most common form of Gram-positive peritonitis [12, 13], which is associated with a 60% risk of recurrence within 6 months [14].

In the present case, there were three abdominal infections within 80 days of initiating urgent-start PD that were caused by *S. aureus*. Traditionally, PD is delayed for 2 weeks after catheter placement to allow proper wound healing. However, some studies have reported that patients with chronic kidney disease who urgently need to start PD within 2 weeks of catheter insertion (urgent-start PD) experienced comparable outcomes to those who commenced PD more than 2 weeks after catheter insertion (conventional-start PD) [9]. Approximately 123 patients have undergone urgent-start PD at our department with few instances of leakage and infection. Thus, we believe urgent-start PD is safe for patients with chronic kidney disease.

In the present case, the patient’s clinical manifestations and microbiological characteristics met the diagnostic criteria for relapsing peritonitis. Peritoneal infection is a common reason for patients to switch from PD to hemodialysis. According to 2016 ISPD guidelines [7], PD catheters should be removed in patients with relapsing peritonitis. Thus, we advised the patient to change to hemodialysis at the second and third episodes of peritonitis before we identified IE, but she refused. Although the first and second abdominal infections were treated with a full course of antibiotics, the patient still relapsed within about 3 weeks. Therefore, we speculated that there may be some other factors involved in this process. Often, the persistent carrier state, including the body surface and visceral organs, is the most likely explanation. *S. aureus*-related peritonitis mainly occurs in patients in whom *S. aureus* colonizes the nares [15–17], skin, or peritoneal catheter exit site [18–20]. Even one positive nasal culture will increase the risk of *S. aureus*-related peritonitis [15, 21]. However, in the present case, there were no pustules on the skin, and the catheter exit site and nares cultures were negative. In addition, computed tomography of the chest and abdomen did not reveal infection or bowel cancer. However, these cultures were taken during the second peritonitis episode. At that time, the patient had already been treated with antibiotic therapy for the first peritonitis episode. This may have influenced the culture results.

In addition, the patient had a history of congenital heart disease, which is a risk factor for IE. Ventricular septal defects, patent ductus arteriosus, tetralogy of Fallot, and aortic valve abnormalities are recognized as risk substrates for IE [22]; however, transthoracic echocardiography indicated ASD in the present case. ASD caused a shunt of blood flow between the atria. Due to the slow shunt speed, the risk of IE from ASD was negligible, and it did not predispose the patient to IE. Therefore, IE associated with ASD is extremely rare, and only a few previous reports exist in the literature [23, 24]. For this reason, we initially ignored the possibility of IE in the present case.

Hemodialysis is one of the known risk factors for IE; however, PD-induced IE has been rarely reported [25, 26]. The antibacterial activity of peritoneal macrophages and the absence of direct access to the circulatory system may be responsible for this low incidence. Only four cases of IE-related peritonitis have been reported prior to the present case (Table 1). *Staphylococcus* is the most commonly involved bacterial genus. Case 3 [27] and case 4 [28] were caused by *S. aureus*, while case 1 [29] was thought to be caused by *Streptococcus faecalis (S. faecalis)*. Of the four cases, only one had a history of heart disease.

**Table 1:** General information and clinical findings of published cases of IE-related peritonitis in patients undergoing PD

Case/Reference	Age	Sex	Previous peritonitis	Echocardiography	Predisposing factors	Blood culture	PD effluent culture	History of cardiac disease	Treatment
1/[29]	13	F	Yes	IE	Recurrent urinary tract infection	Negative	Negative	None	Vancomycin Gentamicin
2/[30]	77	F	NR	IE	NR	*Streptococcus equinus*	*Streptococcus equinus*	None	Vancomycin
3/[27]	39	F	NR	IE	Vertebral osteomyelitis	*Staphylococcus aureus*	*Staphylococcus aureus*	None	Vancomycin Oxacillin
4/[28]	20	M	NR	Autopsy confirmed IE	Chronic rheumatic valvular disease	*Streptococcus faecalis*	*Streptococcus faecalis*	Chronic rheumatic valvular disease	NR

*F* female, *IE* Infective endocarditis, *M* Male, *NR* Not reported, *PD* Peritoneal dialysis

The diagnosis of IE may be difficult in patients with peritonitis who also have fever, encephalopathy, anemia, or cardiac murmur. In contrast to transesophageal echocardiography, transthoracic echocardiography may not be a good tool to detect vegetation. As a result, IE is often missed. In case 1 [29], endocarditis with a tricuspid valve vegetation was diagnosed 2 months after an episode of *Staphylococcus epidermidis* peritonitis. In case 2 [30], persistent *Streptococcus equinus* bacteremia prompted a further search for the source of infection, and finally, a vegetation on the aortic valve was detected by transesophageal echocardiography. In case 3 [27], after the onset of oxacillin-sensitive *S. aureus* peritonitis, transthoracic echocardiography was arranged due to a heart murmur, which showed vegetations above the aortic valve and severe aortic regurgitation. In case 4 [28], mitral valve vegetations were noted on autopsy 1 month after an episode of *S. faecalis* peritonitis with bacteremia. In the present case, the patient was diagnosed with IE on her third admission for relapsing peritonitis by the second transthoracic echocardiography.

The patient in the present case remained free of signs and symptoms of IE, and transthoracic echocardiography revealed no vegetations before PD. However, when other causes of peritonitis relapse were ruled out, particularly at the beginning of the third episode of peritonitis, PD effluent and blood cultures were performed simultaneously and revealed the presence of the same organism, *S. aureus*. The possibility of IE was significantly increased; therefore, a re-examination by transthoracic echocardiography was performed, and a new vegetation was identified. The diagnosis of IE was confirmed by fulfilment of the two main Duke criteria: positive blood cultures and a new vegetation observed on echocardiography. The diagnosis of IE also emphasizes the importance of blood cultures; however, we neglected to perform blood cultures during the first and second episodes of peritonitis, which was one of the main reasons for the delayed diagnosis.

The timing and cause of IE were unclear in the present case. We speculate that the first episode of peritonitis was the cause of transient bacteremia, with IE as the result. Due to the short duration of treatment of the abdominal infection with antibiotics and the fact that the antibiotics were administered via intraperitoneal injection, which meant that the concentration of antibiotics in the blood was lower than it would have been if administered via intravenous injection, IE was not completely cured. The bacteria entered the bloodstream and were transported to the abdominal cavity, resulting in repeated recurrence of abdominal infection. A previous study showed that mesothelial cells can ingest *S. aureus*. The ingested bacteria proliferate abundantly within mesothelial cells and may be subsequently released [31]. We therefore extended the duration of intraperitoneal antibiotic administration and added the intravenous antibiotics piperacillin and tazobactam according to antibiotic susceptibility. After 1 month of antibiotic treatment, the cardiac vegetation disappeared, and peritonitis has not recurred since the repair surgery. This also confirmed our hypothesis that cardiac IE was the cause of peritonitis recurrence.

In summary, to the best of our knowledge, this is the first report of ASD associated with recurrent peritonitis. PD rarely causes IE, and IE associated with ASD is also extremely rare. However, when a patient with ASD undergoing PD develops peritonitis, especially relapsing peritonitis, the possibility of IE is significantly increased. Once abdominal infection occurs, an adequate course of effective antibiotic treatment is necessary. In addition, ASD repair surgery may be an important contributing factor in preventing peritonitis recurrence.

## Data Availability

All data supporting the case are included in the manuscript.

## Abbreviations

PD: Peritoneal dialysis
ASD: Atrial septal defect
IE: Infective endocarditis
CAPD: Continuous ambulatory peritoneal dialysis
